# Robotic portal resection for mediastinal tumours: a prospective observational study

**DOI:** 10.1186/s13019-024-02660-8

**Published:** 2024-03-26

**Authors:** Wei Gan, Mu-Zi Yang, Zi-Hui Tan, Chu-Long Xie, Tian-Yu Sun, Hao-Xian Yang

**Affiliations:** 1 Department of Thoracic Surgery, Sun Yat-sen University Cancer Center, No. 651, Dongfeng East Road, Guangzhou City, Guangdong Province 510060 P.R. China; 2 State Key Laboratory of Oncology in South China, Collaborative Innovation Center for Cancer Medicine, Sun Yat-sen University Cancer Center, Guangzhou, 510060 P.R. China; 3 Guangdong Provincial Clinical Research Center for Cancer, Sun Yat-sen University Cancer Center, Guangzhou, 510060 P.R. China

**Keywords:** Mediastinal tumour, Robotic portal resection, Short-term outcomes

## Abstract

**Background:**

To demonstrate the effectiveness and feasibility of robotic portal resection (RPR) for mediastinal tumour using a prospectively collected database.

**Methods:**

Data from 73 consecutive patients with mediastinal tumours who underwent RPRs were prospectively collected from August 2018 to April 2023. All patients underwent chest and abdominal enhanced computed tomography (CT) and preoperative multidisciplinary team (MDT) discussion. The patients were stratified into two groups based on tumour size: Group A (tumour size < 4 cm) and Group B (tumour size ≥ 4 cm). General clinical characteristics, surgical procedures, and short outcomes were promptly recorded.

**Results:**

All of the cases were scheduled for RPRs. One patient (1/73, 1.4%) was switched to a small utility incision approach because of extensive pleural adhesion. Two patients (2.8%) converted to sternotomy, however, no perioperative deaths occurred. Most of the tumours were located in the anterior mediastinum (51/73, 69.9%). Thymoma (27/73, 37.0%) and thymic cyst (16/73, 21.9%) were the most common diagnoses. The median diameter of tumours was 3.2 cm (IQR, 2.4–4.5 cm). The median total operative time was 61.0 min (IQR, 50.0–90.0 min). The median intraoperative blood loss was 20 mL (IQR, 5.0–30.0 ml), and only one patient (1.4%) experienced an intraoperative complication. The median length of hospital stay was 3 days (IQR, 2–4 days). Compared with Group A, the median total operative time and console time of Group B were significantly longer (*P* = 0.006 and *P* = 0.003, respectively). The volume of drainage on the first postoperative day was greater in group B than in group A (*P* = 0.013).

**Conclusion:**

RPR is a safe and effective technique for mediastinal tumour treatment, which can expand the application of minimally invasive surgery for the removal of complicated mediastinal tumours.

**Supplementary Information:**

The online version contains supplementary material available at 10.1186/s13019-024-02660-8.

## Introduction

Mediastinal tumours represent a variety of tumours, such as thymomas, teratomas, thyroid diseases, bronchogenic cysts, and neurogenic tumours [1, 2]. Currently, surgery with curative intent is the primary choice for most mediastinal tumours [3]. Traditional surgical approaches include median sternotomy or lateral thoracotomy; however, patients experience extensive trauma and slow recovery from surgery [3]. With the development of minimally invasive surgery (MIS), video-assisted thoracic surgery (VATS) has been accepted for the treatment of mediastinal tumours in selected cases [4]. However, VATS requires a deep learning curve for mediastinal tumour resection and has only been performed in experienced centres with large volumes, which has hindered its use and expansion [5].

In recent years, robot-assisted thoracoscopic surgery (RATS) has raised the interest of surgeons in the treatment of mediastinal tumours with the advantages of three-dimensional visualisation and small-wristed instruments, making fine operations possible as a minimally invasive approach [6–8]. However, the results of the previous studies on robotic mediastinal tumour resection were retrospective [6, 9]. Moreover, studies on robotic mediastinal tumour resection differ significantly in terms of surgical technique and different mediastinal tumour types [10, 11]. Therefore, we believe that more studies are essential to define robotic surgical techniques, their feasibility, and outcomes in mediastinal tumour resection, especially when using prospectively collected data. Our previous studies demonstrated that the robotic portal approach using CO_2_ insufflation could flatten the learning curves and have satisfactory short-term outcomes in lung resections for lung cancer patients [12–15], and was feasible in resection of the challenging mediastinal tumour in technique point of view from a case report [16]. Herein, we designed a prospective observational study that included a series of consecutive patients with mediastinal tumours who underwent robotic portal resections (RPRs) by a single surgical team, with the purpose of further determine the effectiveness and feasibility of the robotic portal approach for minimally invasive resection of a variety of mediastinal tumours in a real-world practice by a single robotic surgical team, and to provide a comprehensive overview of its utility across different mediastinal regions. The prospective nature and the variety of tumour types may be valuable to clinical practice.

## Patients and methods

### Patient selection

We used a prospectively collected database of robotic thoracic surgeries, as described elsewhere [13]. This study was approved by the Institutional Review Board of Sun Yat-sen University Cancer Center on 11 April 2023. All surgeries were performed by a team led by a senior surgeon. The requirement for informed consent was waived because no additional interventions were administered to the patients beyond standard medical care. Patients were included if they met the following criteria: (i) primary mediastinal tumours and (ii) suitability for RATS by a multidisciplinary team (MDT) discussion.

Review the previously published literature and refer to their grouping criteria [17, 18], all included patients were stratified into two groups to compare perioperative outcomes: the diameter of the tumours was less than 4 cm (Group A) and the diameter of the tumours was 4 cm or greater than (Group B).

### Data collection

A comprehensive case report form was designed for each case as described previously [13]. General clinical characteristics, such as sex, age, tumour location, tumour size (radiologic size), smoking history, and comorbidities were recorded preoperatively. The detailed process and time consumption, which reflected the key steps of the operation, were simultaneously recorded. In addition, blood loss, intraoperative complications, and reasons for conversion (if occurring) were recorded during each operation. The pathological type and stage, drainage on postoperative day one, chest tube duration, postoperative complications, length of postoperative hospital stay, and postoperative costs were recorded. Thymomas were classified according to the World Health Organization (WHO) histological classification and staged according to the Masaoka-Koga staging system [19, 20]. Perioperative complications (Grade II or higher) were recorded and classified according to the Clavien–Dindo Classification System [21].

Surgical time, including docking time, console time, and total operative time, was analysed to assess surgical efficiency. Perioperative parameters, such as blood loss during surgery, conversion to an open procedure, chest tube duration, length of hospital stay, and perioperative complications were analysed to assess the safety and effectiveness of the operation.

### Surgical techniques

**Fig. 1:**
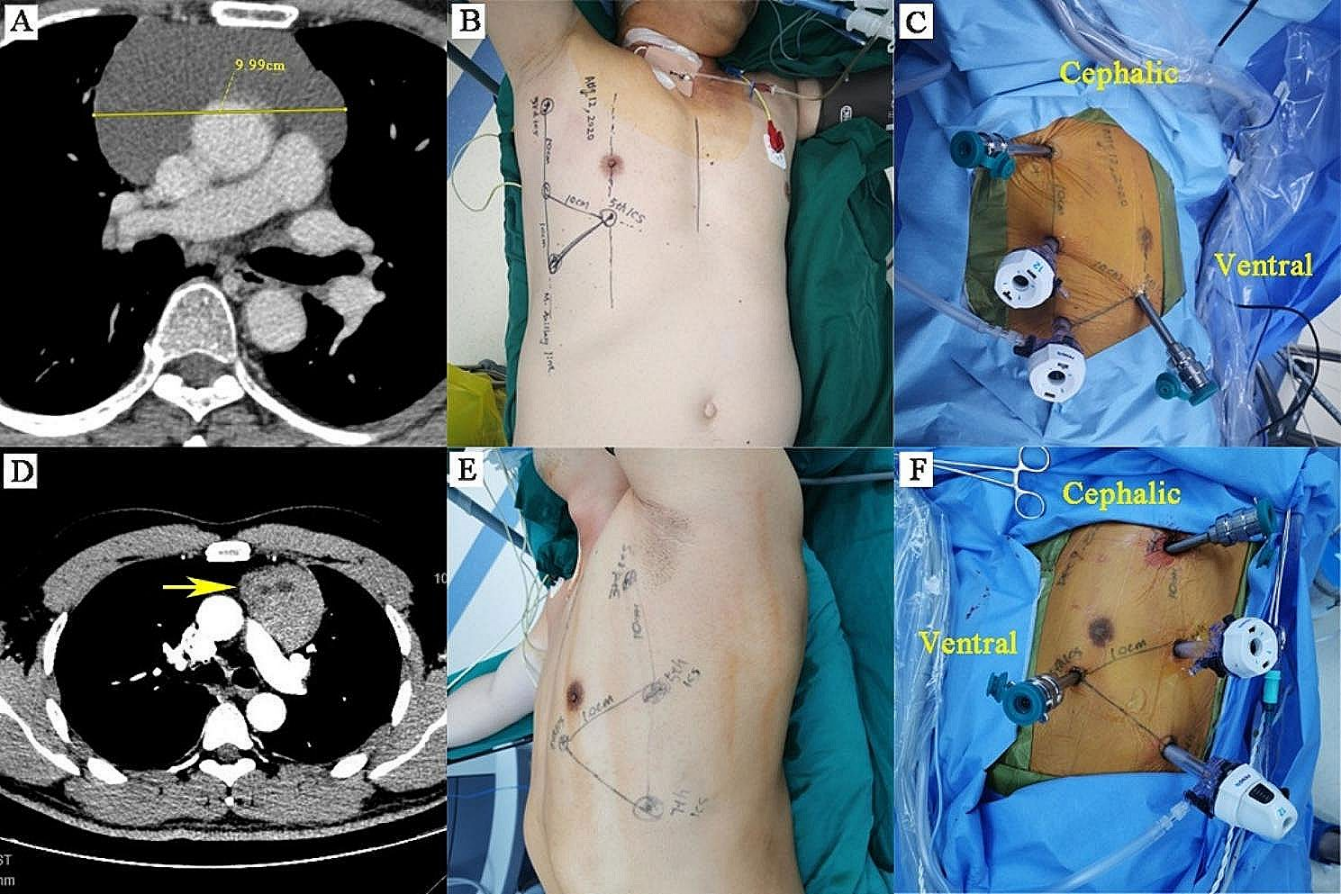
Chest computed tomography (CT) image, operating position, and port location for robotic anterior and superior mediastinal tumour surgery. (**A-C**) Semi-lateral position with 45 degrees elevation (right approach). (**D-F**) Semi-lateral position with 45 degrees elevation (left approach). Two 8-mm ports were used for the robotic instruments (right for an ultrasonic scalpel and electric hook, left for a fenestrated bipolar forceps to assist in exposing the surgical field), and one 12**-**mm (for the Da Vinci Si system)/8**-**mm (for the Da Vinci Xi system) port was used for the robotic camera. A 12**-**mm trocar was placed in the seventh intercostal space on the midaxillary line as an assistant port. Each adjacent port was made 8–10 cm apart to avoid obstruction between instruments, and CO_2_ insufflation was set at 6–8 mmHg

**Fig. 2:**
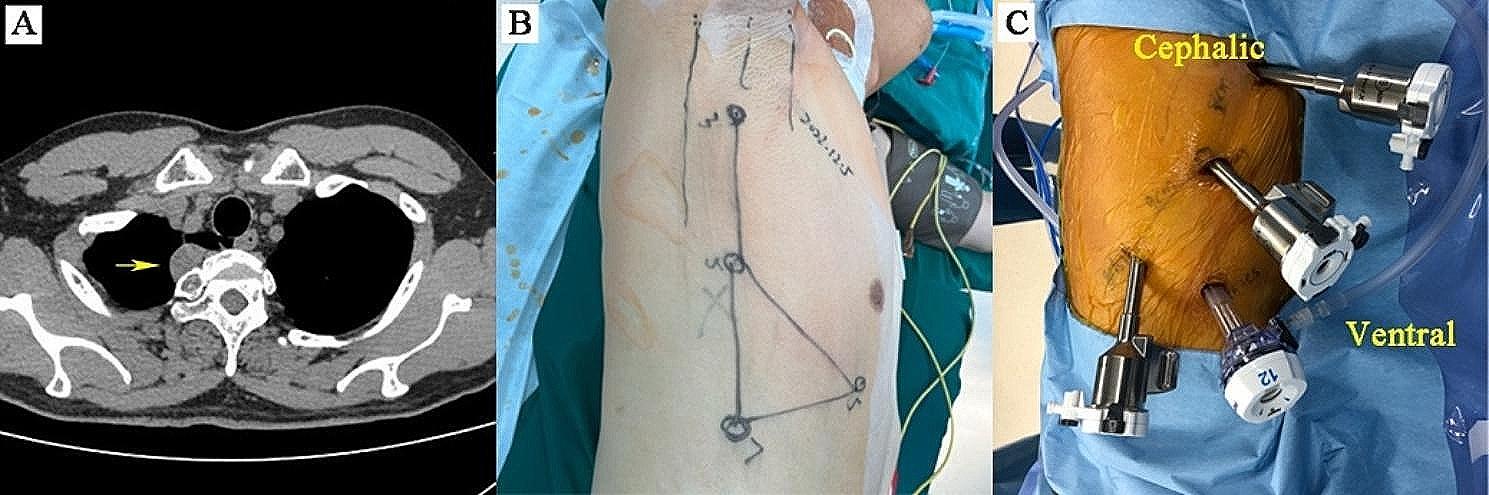
Chest CT image, operating position, and port location for robotic posterior mediastinal tumour surgery. (**A**) The preoperative computed tomographic scan revealed a suspected schwannoma on the right thoracic paravertebral side. (**B, C**) Lateral decubitus position

**Fig. 3:**
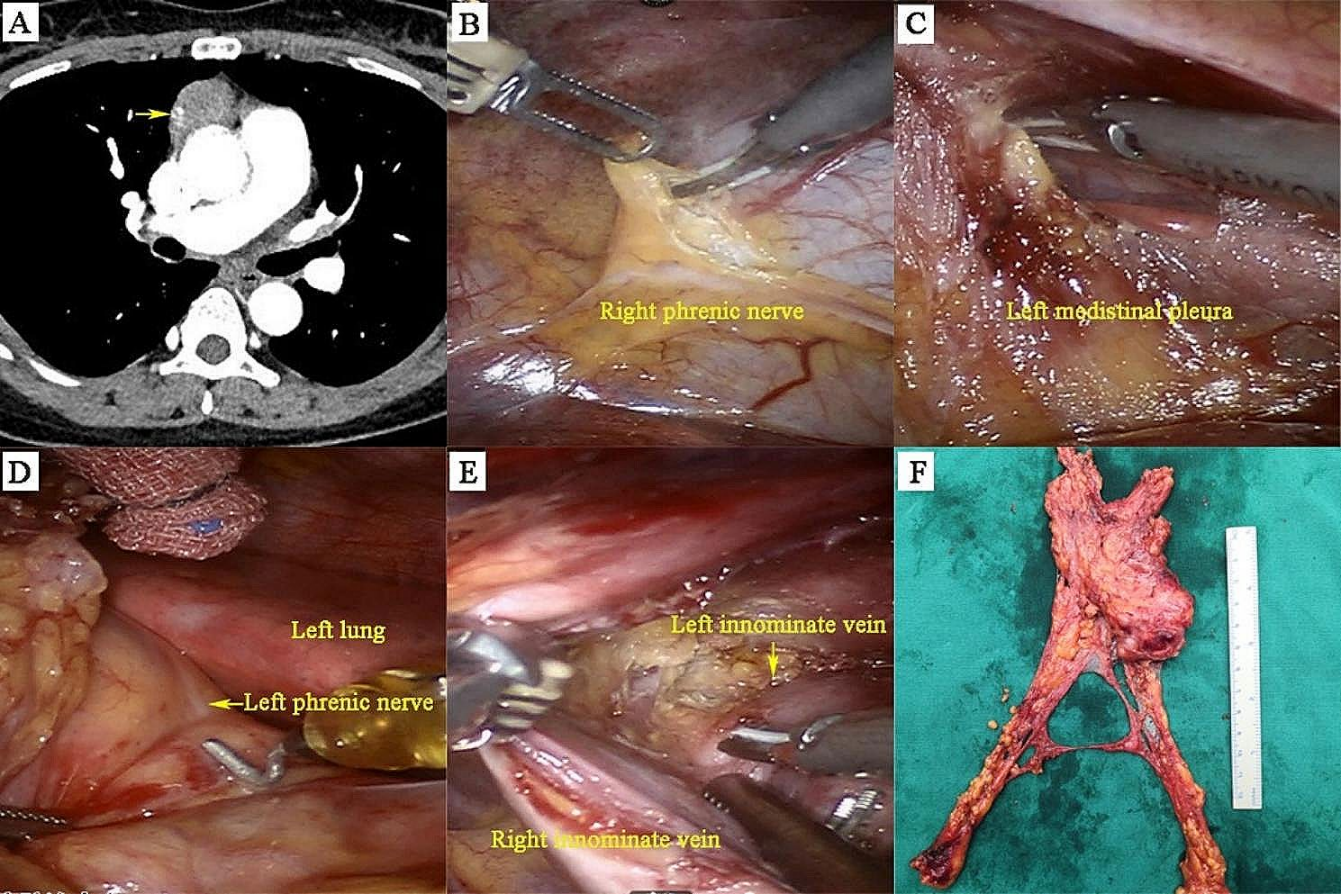
The surgical practice of extended thymectomy. (**A**) The preoperative computed tomographic scan revealed a suspected thymoma (3.0 × 2.7 cm). (**B**) Intraoperative images of right phrenic nerve. (**C, D**) Left mediastinal pleura and left phrenic nerve. (**E**) Region of the upper pole of the thymus and brachiocephalic vein. (**F**)Postoperative gross specimen

All surgeries were performed by the same surgical team using a da Vinci Si/Xi system (Intuitive Surgical, Inc.). We preferred RPRs with CO_2_ insufflation using the three arms of the robotic system except for two cases that adopted the four-arm portal procedure. General anaesthesia and a double-lumen endotracheal tube were used in all cases. For anterior and superior mediastinal tumours, the patient was placed in a supine position with 45° elevation on the affected side (semi-lateral decubitus position). The detailed methods for determining the surgical position and port setting are shown in Fig. 1. If the centre of the mass was on the right or middle side of the thorax, a right-sided approach was used; otherwise, a left-sided approach was used (Fig. 1). The lateral decubitus position was used for posterior mediastinal tumours (Fig. 2). Two 8-mm ports were used for the robotic instruments (right for an ultrasonic scalpel and electric hook, left for fenestrated bipolar forceps to assist in exposing the surgical field), and one 12-mm (for the Da Vinci Si system)/8-mm (for the Da Vinci Xi system) port was used for the robotic camera. A 12-mm trocar was placed in the seventh intercostal space at the midaxillary line as an assistant port. Each adjacent port was placed 8–10 cm apart to avoid obstruction between instruments, and CO_2_ insufflation was set at 6–8 mmHg. In our study, complete resection of the tumour alone or thymectomy is sufficient for benign tumours such as a thymic cyst or neurogenic tumour. In patients diagnosed with thymoma, extended thymectomy is required to reduce the risk of postoperative recurrence, following the National Comprehensive Cancer Network (NCCN) guidelines for Thymomas and Thymic Carcinomas [22]. The scope of surgical resection was well defined: both the upper and lower poles were in continuity along with all mediastinal adipose tissue within the borders of the phrenic nerves, diaphragm, and cervical border of the anterior mediastinum cephalad to the innominate vein. If the tumour invades adjacent structures, such as the pericardium, phrenic nerve, pleura, or lung tissue, it should also be excised; however, bilateral phrenic nerve injury should be avoided [5, 23]. To illustrate the approach of the robotic portal procedure, a representative case is presented in Fig. 3 along with a surgical video online only (Video 1). After resection, specimens were removed from the endoscopic pouches through an enlarged assistant port in patients who underwent complete portal procedures. A 24 Fr chest tube was inserted through the camera port for drainage, and simple closed thoracic drainage was primarily employed without routine negative pressure suction.

**Fig. 4:**
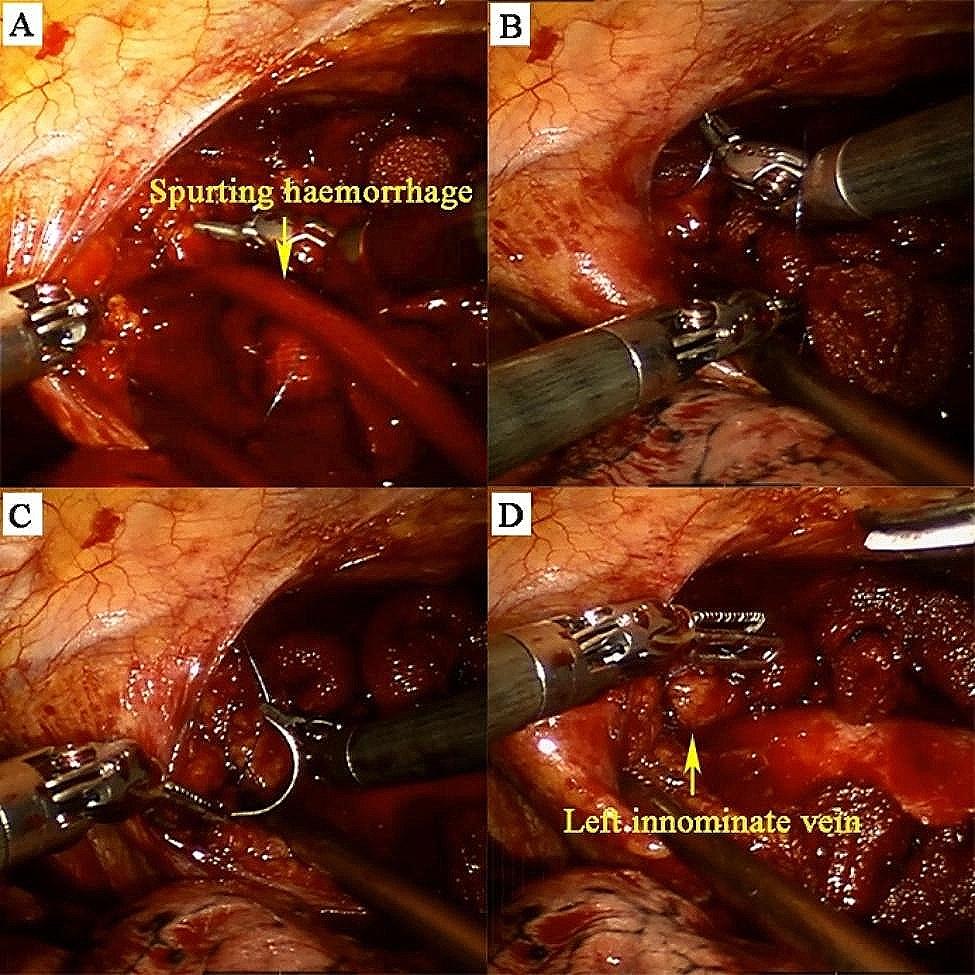
Representative case of left innominate vein injury and repair. (**A**) Left innominate vein injury and bleeding. (**B-D**) The bleeding was stopped by suture. A 48-year-old male, preoperative CT scan revealed a mass in the anterior mediastinum with clear boundary, about 40 mm×34 mm in size, indicating a high probability of thymoma. After eliminating contraindications, a robotic portal thymectomy was performed. During the procedure, the left innominate vein injury occurred and the bleeding site was near the confluence of the superior vena cava. The bleeding site was compressed with a gauze piece for hemostasis and then closed with an 8-character pattern suturing using a 4−0 prolene suture

It is well known that the removal of the upper pole of the thymus during extended thymectomy is very difficult and prone to left innominate vein injury and intraoperative bleeding. And it is extremely challenging to stop bleeding under minimally invasive surgery. With the advantages of flexible instruments and high-definition view, robotic surgery may facilitate the rapid and safe discontinuation of intraoperative bleeding to avoid conversion to open surgery. Here, we present a case to assist in illustrating the advantages of robotic surgery for intraoperative hemostasis (Fig. 4). The detailed operation was shown in the surgical video provided by us (Video 2).

### Pathological diagnoses and postoperative management

The resected surgical specimens were routinely sent for pathological examination and Hematoxylin and Eosin (H&E) staining and Immunohistochemistry (IHC) are pivotal techniques. Histological typing was determined according to the classification system of the World Health Organization [19]. Thymomas and thymic carcinoma were staged according to the Masaoka-Koga staging system [20].

Postoperative care primarily involves managing pain, preventing infections, and maintaining good respiratory function. If the postoperative drainage is less than 300 milliliters and without air leakage, and the chest X-ray doesn’t show obvious effusion, atelectasis, or pneumothorax, the removal of the chest tube can be considered. Patients with benign tumours do not routinely require extensive follow-up or repeated examinations. For patients diagnosed with malignant tumours, regular follow-ups and check-ups are necessary to monitor for any recurrence. Chest CT every 6 months for 2 years, then annually for 10 years for thymoma and annually for 5 years for thymic carcinoma, respectively [24].

### Statistical analysis

Statistical analyses were performed using the IBM SPSS Statistics (version 23.0; IBM Corp.). Categorical data are presented as numbers and percentages, and continuous data are presented as medians and interquartile ranges (IQR). Statistical comparisons between the groups were performed using the Wilcoxon rank-sum test for continuous variables and Pearson’s chi-square or Fisher’s exact test for categorical data. A two-sided *P* < 0.05 was considered statistically significant.

## Result

### Clinicopathological characteristics of patients

Consequently, 73 consecutive patients (28 men and 45 women) with mediastinal tumours who were scheduled for PRRs between August 2018 and April 2023 were enrolled. The clinicopathological characteristics of the patients are summarised in Table S1. The median age of all patients was 47 years (IQR, 33–59 years). Most of the tumours were from the anterior and superior mediastinum (63/73, 85.9%), and the median size was 3.2 cm (IQR, 2.4–4.5 cm). Most patients were asymptomatic (52/73, 71.2%), and no myasthenia gravis was identified in the entire cohort. Unsurprisingly, the most common tumours originated from the thymus, including thymomas (27/73, 37.0%) and thymic cysts (16/73, 21.9%). Of the 27 patients with thymomas, 19 (70.5%) had Stage I disease.

The clinicopathological characteristics of Groups A and B are summarised in Table 1. There was a significant difference in tumour diameter between the two groups (*P* < 0.001), but no significant differences in age (*P* = 0.212), gender (*P* = 0.461), smoking status (*P* = 0.223), comorbidities (*P* = 0.624), tumour location (*P* = 0.479), surgical approach (*P* = 0.529) and histology (*P* = 0.202). Patients in Group B were more likely to develop symptoms because of the large tumours, but the difference was not statistically significant (*P* = 0.189).

**Table 1:** Clinicopathological characteristics of Group A and Group B

Characteristics	Group A^a^ (*N* = 45)	Group B^b^ (*N* = 28)	*P* value
Age (years)			
Median (IQR)	48 (35, 59)	39 (32, 59)	0.212
Gender			0.461
Male	15 (33.3%)	12 (42.9%)	
Female	30 (66.7%)	16 (57.1%)	
Smoking			0.223
Current	0	1 (3.6%)	
Quit	5 (11.1%)	4 (14.3%)	
Never	40 (88.9%)	23 (82.1%)	
BMI (kg/m²)			0.532
< 18.5	3 (6.7%)	2 (7.1%)	
18.5–24.9	30 (66.7%)	17 (60.7%)	
25.0–29.9	11 (24.4%)	6 (21.4%)	
> 30.0	1 (2.2%)	3 (10.7%)	
Symptom			0.189
Asymptomatic	36 (80.0%)	16 (57.1%)	
Cough	4 (8.9%)	3 (10.7%)	
Chest pain	3 (6.7%)	4 (14.3%)	
Chest tightness	1 (2.2%)	3 (10.7%)	
Other	1 (2.2%)	2 (7.1%)	
Tumour location			0.479
Left anterior	11 (24.4%)	9 (32.1%)	
Right anterior	8 (17.8%)	7 (25.0%)	
Anteromedian	13 (28.9%)	3 (10.7%)	
Superior	6 (13.3%)	6 (21.4%)	
Left posterior	4 (8.9%)	2 (7.1%)	
Right posterior	3 (6.7%)	1 (3.6%)	
Tumour size (cm)			< 0.001
Median (IQR)	2.6 (1.8, 3.0)	5.0 (4.2, 6.5)	
Comorbidities			0.624
Yes	15 (33.3%)	11 (39.3%)	
No	30 (66.7%)	17 (60.7%)	
Approach			0.529
Left	15 (33.3%)	12 (42.9%)	
Right	28 (62.2%)	16 (57.1%)	
Subxiphoid	2 (4.4%)	0	
Histology			0.202
Thymoma	17 (37.8%)	10 (35.7%)	
Thymic cyst	13 (28.9%)	3 (10.7%)	
Schwannoma	6 (13.3%)	3 (10.7%)	
Bronchial cyst	3 (6.7%)	2 (7.1%)	
Teratoma	1 (2.2%)	2 (7.1%)	
Thymic hyperplasia	1 (2.2%)	2 (7.1%)	
Thymic SCC	1 (2.2%)	1 (3.6%)	
Castleman disease	0	2 (7.1%)	
Mediastinal cyst	2 (4.4%)	0	
Other	1 (2.2%)	3 (10.7%)	

^a^Group A, defined as the diameter of the tumour was less than 4 cm

^b^Group B, defined as the diameter of the tumour was 4 cm or greater than

IQR, interquartile ranges. SCC, squamous cell carcinoma

Other, Neuroendocrine tumour, Solitary fibrous tumour, Parathyroid cyst, Diffuse large B cell lymphoma

### General perioperative outcomes for the entire cohort

The majority of patients (72/73, 98.6%) underwent PRRs as scheduled, but one patient switched to a small utility incision approach because of extensive pleural adhesions. The median total operative time was 61.0 min (IQR, 50.0–90.0 min). For the entire cohort, the median blood loss was 20 mL (IQR, 5.0–30.0 mL). Only one patient (1.4%) experienced intraoperative complications (left innominate vein injury); however, the injury was sutured using a robotic procedure without thoracotomy conversion. Two patients (2.7%) underwent active conversion to sternotomy because the tumours were found to involve the left innominate vein during surgery. Besides, three patients (4.1%) concurrently underwent lung wedge resections and one patient accepted partial pericardial resection due to local invasion. The median drainage on the first postoperative day was 100.0 mL (IQR, 37.5–180.0 mL) and the median chest tube duration was 2 days (IQR, 2–3 days). The median length of hospital stay was 3 days (IQR, 2–4 days), while the median cost was 9131.9 USD (IQR, 8546.2–9606.7 USD). Postoperative complications were developed in seven (9.6%) patients, including one patient with chylothorax. The sole case of postoperative chylothorax was cured by conservative therapy but not surgery. We initiated treatment with fasting and total parenteral nutrition (TPN) to support the patient and the chylothorax was cured after 4 days of care. No perioperative deaths or secondary operations occurred in the entire cohort. The perioperative outcomes are summarised in Table S2.

### Outcome comparison between Group A and Group B

Since mediastinal tumours represent a variety of tumour types, we further compared the operative outcomes between Groups A and B based on tumour size. The data suggested that the median total operative time and console time of Group B were significantly longer than that of Group A (*P* = 0.006 and *P* = 0.003, respectively). The volume of drainage was greater in Group B than in Group A on the first postoperative day (*P* = 0.013). The amount of intraoperative blood loss, chest tube duration, length of hospital stay, intraoperative complications, postoperative complications, and costs were comparable between the two groups (Table 2). Two cases in Group B were converted to open surgery but none in Group A (*P* = 0.144).

**Table 2:** Comparison of perioperative outcomes between the two groups

Variables	Group A^a^ (*N* = 45)	Group B^b^ (*N* = 28)	*P* value
Total operation time^c^ (min), median (IQR)	56.0 (46.0, 75.5)	85.5 (59.8, 95.3)	0.006
Docking time (min), median (IQR)	5.0 (5.0, 7.5)	6.0 (5.0, 7.5)	0.262
Console time^d^ (min), median (IQR)	28.0 (19.0, 42.0)	51.5 (27.0, 65.5)	0.003
Intraoperative blood loss (mL), median (IQR)	10.0 (5.0, 30.0)	20 (10.0, 45.0)	0.069
Intraoperative complications, n	0	1 (1.4%)	0.384
Intraoperative invasion, n	1 (1.4%)	3 (4.2%)	0.154
Lung	0	3 (4.2%)	
Pericardium	1 (1.4%)	0	
Conversion, n	0	2 (2.8%)	0.144
Drainage on POD 1 (mL), median (IQR)	100.0 (20.0, 140.0)	140.0 (72.5, 256.3)	0.013
Chest tube duration (days), median (IQR)	2 (2, 3)	2 (2, 3)	0.196
Length of hospital stay (days), median (IQR)	2 (2, 3.5)	3 (2, 5)	0.157
Postoperative complications, n	5 (6.8%)	2 (2.7%)	> 0.999
Hypokalemia	2 (2.8%)	0	
Air leakage	0	1 (1.4%)	
Chylothorax	1 (1.4%)	0	
Dyspnea	0	1 (1.4%)	
Hypotension	1 (1.4%)	0	
Pleural effusion	1 (1.4%)	0	
Cost ($), median (IQR)	9037.2 (8490.6, 9551.3)	9216.4 (8617.2, 9853.9)	0.207

^a^Group A, defined as the diameter of the tumour was less than 4 cm

^b^Group B, defined as the diameter of the tumour was 4 cm or greater than

^c^Defined as the time of skin to skin. Patients with conversion were excluded

^d^Defined as the time of operating console. Patients converted to open surgery were excluded

POD 1, postoperative day one. IQR, interquartile ranges

### Follow up

Patients with benign tumours do not routinely require extensive follow-up or repeated examinations. For patients diagnosed with malignant tumours, regular follow-ups, and check-ups are necessary to monitor for any recurrence. There were 27 thymomas, two thymic carcinomas, one mediastinal lymphoma, and one neuroendocrine tumour. The follow-up period ranged from 5 to 60 months, with a median follow-up of 24 months. No patients presented radiological evidence of recurrence, and no patient died.

## Discussion

Since the first report of robotic thymectomy in 2001 [4], RATS has been used to treat mediastinal tumours in some large-volume medical centres [25, 26]. However, previous studies were retrospective [6, 9], and the surgical approach and technique varied among different centres from a technical perspective. Therefore, further studies are warranted to define the robotic surgical technique and its feasibility and outcomes in mediastinal tumour resection using prospectively collected data. We prospectively included 73 consecutive patients with mediastinal tumours who underwent RPRs, in which a complete robotic portal procedure with CO_2_ insufflation was used as the preferred approach. The outcomes demonstrated that this procedure is safe and feasible for mediastinal tumour resection, even for selected complicated tumours. The findings of this study may help to flatten the learning curve and broaden the application of a minimally invasive approach for the surgical treatment of mediastinal tumours and benefit patients by avoiding extensive trauma. The prospective nature of this study lends itself to its reliability and makes it meaningful for clinical practice.

Since Cerfolio et al. introduced the complete portal robotic surgical technique through a lobectomy series in 2011, it has been rapidly promoted and applied in thoracic surgery [27]. In this study, all operations were performed by one surgical team and initiated with complete portal robotic surgery with CO_2_ insufflation, except for one case in which a small utility incision was adopted because of the extensive pleural adhesion that hindered the port setting. We made a small utility incision to facilitate adhesion release and enabled the completion of the port setting. The complete-portal robotic surgical technique provides many advantages over traditional robotic thoracic surgery with a utility incision. First, CO_2_ insufflation provides a better view of the surgical field, extending the endoscopic field by lowering the diaphragm and compressing the lung [13, 15, 28]. This is particularly important for mediastinal tumour resection in remote and narrow spaces. Second, the complete portal robotic procedure described in this study can help avoid interference between surgical instruments and reduce the difficulty of surgery, thereby reducing the requirement for surgical assistance. Only one junior doctor was qualified to perfectly coordinate with the surgeon to complete the operation. In addition, for some small tumours, the specimen can be removed directly from the assistant port without extending the incision, which is conducive to postoperative pain management and rapid recovery. We believe that these advantages in favour of generalisation can help facilitate the widespread use of this technique in clinical practice.

Short-term outcomes, such as perioperative complications, are essential parameters to evaluate the safety and feasibility of the surgical technique [17, 29]. In this study, only two patients underwent sternotomy, both of which were converted intentionally because of suspected invasion of the innominate veins during intraoperative exploration; however, no operative deaths occurred, indicating the safety and feasibility of our procedures. Similar outcomes were also reported in other studies [6, 9, 25]. Currently, resection of mediastinal tumours involving major vessels using MIS is challenging, and median sternotomy remains the primary choice for patients with these tumours [30]. One advantage of RATS is the quick and safe discontinuation of intraoperative bleeding to avoid conversion to open surgery [30]. Using the fenestrated forceps of the robot, surgeons can directly grab the crevasses of blood vessels to temporarily stop bleeding. Subsequently, surgeons could deal with injured vessels by suturing or other management techniques, which could avoid conversion to sternotomy or thoracotomy in some cases [30]. In our study period, only one patient had intraoperative bleeding caused by a left innominate vein injury; however, the bleeding point was successfully sutured without conversion owing to the articulated wrist for suturing. Although there was no control group, the fact that there were no emergency conversion in this study indicates that the robotic procedure was superior to the previously reported VATS procedure which showed a much higher unplanned conversion rate [31].

Previous studies have suggested that it is time-consuming to dock a robot and exchange instruments during surgery [17, 32, 33]. The proficiency of the assistant and the perfect cooperation of the scrub nurse could effectively reduce these time expenses. With the application of the complete portal robotic surgical technique and perfect cooperation between the assistant and operator, both the docking time and total operation time in this study were much shorter than those in previous studies [6, 8]. The console time is an essential index that reflects the complexity of the surgery and intraoperative conditions. However, many cohort studies lack such data because of their retrospective nature [6, 9]. However, our experience revealed that the console time could be reduced compared to VATS. The results of this study are important for the application and expansion of this technique. During the study period, we also performed other robotic procedures, such as robotic lobectomies and oesophagectomies, which may have also contributed to shortening the docking and console times in this study [13–15]. Compared to previous studies, the rest of the perioperative outcomes in our study were comparable or even better, including intraoperative blood loss, postoperative complications, chest tube, drainage, and duration and length of hospital stay [6, 9]. The results of our study confirm the safety and reliability of RPR for mediastinal tumours.

There are various types of mediastinal tumours [34]. The pathology in the anterior and superior mediastinum was mainly originated from the thymus, whereas that of the posterior mediastinum was mainly neurogenic. Owing to the different anatomic structures around the tumours and different oncological characteristics, a large difference may exist in terms of surgical outcomes between these tumours. In addition, the selection of suitable patients has traditionally been the critical factor to the success of robotic surgery, especially those with large sizes and complex anatomical positions has always been considered as a contraindication for robotic surgery[18, 35]. To analyzed the effect of tumour size on perioperative outcomes, all enrolled patients were divided into two groups according to tumour size, with a cutoff of 4 cm. The data suggested that Group B (tumours over 4 cm) had longer operative time, more intraoperative blood loss, longer chest tube drainage, more cost, and longer hospital stay. Moreover, Group B had more perioperative complications, and both the cases that were converted to sternotomy were from Group B. These outcomes are not surprising given the malignant nature of thymoma, with a larger tumour diameter indicating a higher degree of malignancy and invasiveness, more likely to invade adjacent important structures, such as pericardium and innominate vein [19]. The traditional view is that when the tumour is larger than 4 cm, it is not suitable for minimally invasive robotic surgery, and open surgery should be employed [17, 36]. In this study, however, with the assistance of a robot, most patients in Group B (tumours over 4 cm) were able to avoid thoracotomy, thereby reducing surgical trauma and accelerating postoperative recovery. These results showed that with the help of robots, the indications for minimally invasive surgery could be expanded. But we should keep in mind that the size of the tumour is not the sole factor to consider in the surgical approach, but the tumour’s relationship with surrounding tissues, such as vascular structures is also crucial to determine if MIS is feasible. For example, some large malignant tumours invade the surrounding vital structures and require neoadjuvant therapy, and the subsequent treatment depends on the response of tumours to the neoadjuvant therapy, the relationship of the tumours with vital structures, and the patients’ physical performance.

Limitations also need to be considered when interpreting our data. First, the sample size was not large enough to conduct subgroup analyses based on different pathological diagnoses, and we will continue to collect additional cases from the prospective database. Second, this was an observational study that focused on the technical point of view but lacked control over other surgical techniques.

In conclusion, our results demonstrated that RPR was safe and effective in the surgical treatment of mediastinal tumours and may help expand the indications of MIS and benefit selected complicated patients by avoiding extensive trauma due to large incisions.

### Electronic supplementary material

Below is the link to the electronic supplementary material.

Supplementary Material 1

Supplementary Material 2

Supplementary Material 3

Supplementary Material 4

## Data Availability

The authenticity of this article has been validated by uploading the key raw data onto the Research Data Deposit platform (www.researchdata.org.cn), with the approval RDD number as RDDA2023427347.

## Abbreviations

RATS: Robot-assisted thoracoscopic surgery
RPR: Robotic portal resection
VATS: Video-assisted thoracic surgery
MIS: Minimally invasive surgery
WHO: World Health Organization
IQR: Interquartile ranges
NCCN: National Comprehensive Cancer Network
USD: US dollars
CO_2_: Carbon dioxide
