# Supplementary material for: Robotic portal resection for mediastinal tumours: a prospective observational study
Source: J Cardiothorac Surg. 2024 Mar 26;19:155. doi: 10.1186/s13019-024-02660-8 (PMC10964499; doi:10.1186/s13019-024-02660-8)
Supplement: Supplementary file 3 — Supplementary Material 3 [file 13019_2024_2660_MOESM3_ESM.docx]

| **Table S1. Clinicopathological characteristics of patients** | |
| --- | --- |
| Characteristics | Number of Patients (%) |
| Age (years) |  |
| Median (IQR) | 47 (33, 59) |
| Gender |  |
| Male | 27 (37.0%) |
| Female | 46 (63.0%) |
| Smoking |  |
| Current | 1 (1.4%) |
| Quit | 9 (12.3%) |
| Never | 63 (86.3%) |
| BMI (kg/m²) |  |
| < 18.5 | 5 (6.8%) |
| 18.5 – 24.9 | 47 (64.4%) |
| 25.0 – 29.9 | 17 (23.3%) |
| > 30.0 | 4 (5.5%) |
| Symptom |  |
| Asymptomatic | 52 (71.2%) |
| Cough | 7 (9.6%) |
| Chest tightness | 7 (9.6%) |
| Chest pain | 4 (5.5%) |
| Other | 3 (4.1%) |
| Comorbidities |  |
| Yes | 26 (35.6%) |
| No | 47 (64.4%) |
| Tumour location |  |
| Left anterior | 20 (27.4%) |
| Right anterior | 15 (20.5%) |
| Anteromedian | 16 (21.9%) |
| Superior | 12 (16.4%) |
| Left posterior | 6 (8.2%) |
| Right posterior | 4 (5.5%) |
| Tumour size (cm) |  |
| Median (IQR) | 3.2 (2.4, 4.5) |
| Approach |  |
| Right | 44 (60.3%) |
| Left | 27 (37.0%) |
| Subxiphoid | 2 (2.7%) |
| Histology |  |
| Thymoma | 27 (37.0%) |
| Thymic cyst | 16 (21.9%) |
| Schwannoma | 9 (12.3%) |
| Bronchial cyst | 5 (6.8%) |
| Teratoma | 3 (4.1%) |
| Thymic hyperplasia | 3 (4.1%) |
| Thymic SCC | 2 (2.7%) |
| Castleman disease | 2 (2.7%) |
| Mediastinal cyst | 2 (2.7%) |
| Other | 4 (5.5%) |
| WHO class^a^ |  |
| A | 1 (3.4%) |
| AB | 14 (48.3%) |
| B1 | 3 (10.3%) |
| B2 | 3 (10.3%) |
| B2+B3 | 3 (10.3%) |
| Microscopic thymoma | 1 (3.4%) |
| Metaplastic thymoma | 1 (3.4%) |
| Micronodular thymoma with lymphoid stroma | 1 (3.4%) |
| Masaoka-Koga stage^b^ |  |
| I | 19 (70.4%) |
| IIA | 7 (25.9%) |
| IIB | 1 (3.7%) |
| ^a, b^Only thymomas were included. | |
| IQR, interquartile ranges. | |
| SCC, squamous cell carcinoma. | |
| WHO, World Health Organization | |
| Other, Neuroendocrine tumour, Solitary fibrous tumour, Parathyroid cyst, Diffuse large B cell lymphoma | |
