# Supplementary material for: Robotic portal resection for mediastinal tumours: a prospective observational study
Source: J Cardiothorac Surg. 2024 Mar 26;19:155. doi: 10.1186/s13019-024-02660-8 (PMC10964499; doi:10.1186/s13019-024-02660-8)
Supplement: Supplementary file 4 — Supplementary Material 4 [file 13019_2024_2660_MOESM4_ESM.docx]

| **Table S2. General perioperative outcomes for the entire cohort** | |
| --- | --- |
| Variables | Value |
| Total operation time^a^ (min), median (IQR) | 61.0 (50.0, 90.0) |
| Console time^b^ (min), median (IQR) | 34.0 (20.0, 55.0) |
| Docking time (min), median (IQR) | 6.0 (5.0, 7.0) |
| Intraoperative blood loss (mL), median (IQR) | 20.0 (5.0, 30.0) |
| Intraoperative complications, n |  |
| Left innominate vein haemorrhage, n | 1 (1.4%) |
| Intraoperative invasion |  |
| Lung | 3 (4.1%) |
| Pericardium | 1 (1.4%) |
| Conversion to sternotomy, n | 2 (2.7%) |
| Drainage on POD 1 (mL), median (IQR) | 100.0 (37.5, 180.0) |
| Chest tube duration (days), median (IQR) | 2 (2, 3) |
| Postoperative complications, n | 7 (9.6%) |
| Hypokalemia | 2 (2.7%) |
| Dyspnea | 1 (1.4%) |
| Hypotension | 1 (1.4%) |
| Pleural effusion | 1 (1.4%) |
| Air leakage | 1 (1.4%) |
| Chylothorax | 1 (1.4%) |
| Length of hospital stay (days), median (IQR) | 3 (2, 4) |
| Cost ($), median (IQR) | 9131.9 (8546.2, 9606.7) |
| ^a^Defined as the time of skin to skin. Patients with conversion were excluded. | |
| ^b^Defined as the time of operating console. Patients converted to open surgery were excluded. | |
| POD 1, postoperative day one. IQR, interquartile ranges. | |
